# Generative AI in healthcare: challenges to patient agency and ethical implications

**DOI:** 10.3389/fdgth.2025.1524553

**Published:** 2025-06-18

**Authors:** Scott A. Holmes, Vanda Faria, Eric A. Moulton

**Affiliations:** ^1^ Pediatric Pain Pathway Lab, Department of Anesthesia, Critical Care and Pain Medicine, Boston Children’s Hospital/Harvard Medical School, Boston, MA, United States; ^2^ Department of Anesthesia, Critical Care and Pain Medicine, Harvard Medical School, Boston Children’s Hospital, Boston, MA, United States; ^3^ Department of Psychology, Uppsala University, Uppsala, Sweden; ^4^ Brain and Eye Pain Imaging Lab, Department of Anesthesia, Critical Care and Pain Medicine, Boston Children’s Hospital/Harvard Medical School, Boston, MA, United States; ^5^ Department of Ophthalmology, Boston Children's Hospital/Harvard Medical School, Boston, MA, United States

**Keywords:** agency, artificial intelligence, brain imaging, survey, database

## Abstract

Clinical research is no longer a monopolistic environment wherein patients and participants are the sole voice of information. The introduction and acceleration of AI-based methods in healthcare is creating a complex environment where human-derived data is no longer the sole mechanism through which researchers and clinicians explore and test their hypotheses. The concept of self-agency is intimately tied into this, as generative data does not encompass the same person-lived experiences as human-derived data. The lack of accountability and transparency in recognizing data sources supporting medical and research decisions has the potential to immediately and negatively impact patient care. This commentary considers how self-agency is being confronted by the introduction and proliferation of generative AI, and discusses future directions to improve, rather than undermine AI-fueled healthcare progress.

## Introduction

The integration of generative AI technologies into clinical research creates a chimeric environment that can distort the concept of self-agency from the perspective of the participant/patient and the experimenter/clinician. A chimeric environment refers to a hybrid environment wherein both human derived and synthetically created data are placed in equal weighting to support algorithm development. This is performed on occasion during algorithm training which has been shown to be an efficient way of improving artificial intelligence models, relative to using only non-synthetic data (1, 2). Artificial intelligence reflects the ability of a machine to perform beyond its explicit programming. Algorithms act to: (1) learn the core features that represent an input data set and (2) leverage this reduced set of features and latent space to generate novel products (e.g., text or images). These algorithms have the capacity to deviate regenerated data to extend beyond the original source document(s), to produce novel items that are changed in some fundamental supervised, or unsupervised way. In the context of generative AI, patient agency is a central concern in so much as it relates to the extent to which individuals can make autonomous, informed decisions about their health. Patient agency is being redefined and shaped not only by the accessibility, and interpretability of generative AI models, but also by the degree to which patients can trust, question, and act upon the information provided.

The recent growth of AI technologies in healthcare, based largely on the advent of more capable graphic processor units (GPU) and the digitization of healthcare data, has yielded a double edge sword; at once both enabling models that improve patient care and mitigate medical resource limitations (3), while also decoupling the patient-clinician relationship. This previously transparent interaction has evolved opaque characteristics, directly challenging the concept of patient agency (see (4–6) for review). It is at this point that persons working with generative AI seem to have the largest impact. As we begin to rely more on elements like telemedicine, and remote survey work, factors such as agent authentication are becoming a credible concern towards medical accuracy and accountability. The use of techniques such as generative adversarial networks to produce diagnostic images can create valuable training environments, at times surpassing the use of non-synthetic data (7); however, they may also create environments that extend past healthy human constraints and promote unhealthy lifestyles. We outline relevant concerns regarding the integration of generative AI methods with healthcare regarding patient agency.

## To whom am I speaking with?

The power and capacity of generative AI in terms of large language models is growing rapidly. At the writing of this commentary, Google continues to deploy new iterations of its Gemini program that rivals those of competitors at OpenAI (8) and Claude who also continue the release of revised versions of their core programs. Having been trained on the entirety of text from the internet, these models are highly capable of text-based interface, including being able to provide written prompts, and generating answers, either to describe their response (e.g., explaining a script of code it generated) or for the response itself (e.g., writing an essay). As output is derived from human-based sources, researchers have begun to question, can algorithms such as large language models replace the use of human participants in research (9). Considering that generative models can adapt to text, images, and auditory modalities, the implications for healthcare are immense.

In clinical research, the agent under interest is the person for whom we want to explore a research question and develop our knowledge. This person can be an individual or part of a larger cohort; however, they have unique attributes that address how they experience their condition including their (medical) history, their personal experiences, and how their condition has shaped their perceptions on how their thoughts and actions are self-generated. This, in effect, represents their agency. The application of generative models directly undermines this element of agency by removing the person, or agent, and promoting the viewpoints or biases from their training data. Generative AI methods have the capacity to propagate ethical and gender stereotypes in text-based materials including patient reports, education materials, and patient communication [see (10)]. It has also been argued that generative AI methods could trigger, perpetuate, or exacerbate experiences in vulnerable populations, especially those reliant on such healthcare devices for information (11). Even before the proliferation of generative AI, a cottage industry of survey takers in economically-disadvantaged countries presented concerns regarding the validity of crowd-sourced data from internet-based surveys (12). One study found that approximately 33%–46% of survey takers would leverage the use of large language models in their responses (13) while other more recent research has found issues with up to 96% of their online survey findings (14). This concern has already led to a market of online survey tools meant to counter this influence, such as the Mturk Toolkit from CloudResearch (15). Medical doctors and researchers are turning more towards big data to fuel efforts at precision medicine (16) using online data gathering with the use of surveys for medical research; however, with the proliferation of generative AI this voice may not always be authentic. This remote disconnection produces a void wherein LLM-based approaches can be integrated with existing data collection strategies and begin to control the trajectory of research findings. That is to say, if a research survey was undertaken to address a target cohort (e.g., pediatrics), the findings of such a survey may reflect training data on an established cohort (e.g., adults), therein misleading treatment efforts aimed at integrating the agency of the target cohort. In effect, what we as researchers and clinicians are observing when interfacing with such artificial agents, is a mosaic of integrated and echoed perspectives sewn together through layered neural networks.

### Shifting our diagnostic basis

Generative products are becoming ubiquitous, with access to the engines responsible for their creation coming standard on consumer products such as laptops and phones. The basis of generative AI techniques is to use the knowledge we have gathered to date to train a model that can create new information; information that has to date not existed based on the rules that are composed in the model itself. Intrinsic to this process are the mechanisms through which generative AI algorithms use to extrapolate existing information into novel productions. There is growing use of generative AI methods in healthcare for the creation of novel images of artificial bones (17), and brains (18), though their clinical integration has yet to be formally established. Importantly, as current diagnostic markers are based on normative data sets, the means through which we integrate this information into clinical workflow is imperative to consider.

Normative data sets underlie our understanding of deviance from a healthy standard. It is how we judge neurodevelopment, psychological health, and even brain health. To date, these databases, which have been composed of data collected from real world persons, are largely publicly available and many of us may have even interacted with them at our doctor's office [e.g., DSM-5 (19)]. However, we now face the growing realization that such databases could either be mixed with, or be purely generated from synthetic data sources [see (20)]. As an example, based on financial reasons, it may make more sense to train an algorithm aimed at detecting brain cancer from synthetic databases of MRI, or hybrid databases; however, the origin of such synthetic databases may be from only one sex, or the product of such algorithms may not represent cancerous growths, but rogue attempts at mimicking natural cortical folding patterns in the brain. This proposition is unique from using ML/AI to predict growth curves, for example, and refers rather to generating synthetic text responses or bone x-rays that would then be fed into growth curve models alongside human-derived data. The mere presence of a single data point that was not obtained from a verified human means that such models are hybrid-based and should be interpreted as such. Because such generative algorithms operate on a probability level, they have the inherent risk, for example, of generating data that is not theoretically possible in a human state, or data that does not consider factors such as race (21). Without explicit knowledge of how such algorithms were defined or trained, a clinician could easily be coerced into an improper diagnosis, much as if improper reagents were used to perform a standard blood test. As such, the basis from which we judge informed decisions can be manipulated, in a direction that hinders the clinical process. This chimeric integration of data sources could mean that therapeutic goals are shifted, and disease states are missed.

### Future directions

To date, we have not been labeling data as AI produced vs. “uniquely human.” And why would we? This has not been a concern until recently. Attempts at chimeric databases have largely focused on non-healthcare data, producing images such as flowers. As the capacity of such algorithms grows alongside data storage capacity, it will be critical to isolate synthetic from non-synthetic databases and regulate their clinical integration. In terms of large language models, there are notable modern attempts to evaluate their capacity in clinical settings using evaluations such as CRAFT-MD (Conversational Reasoning Assessment Framework for Testing in Medicine) that highlight the exceptional capacity, but continued deficiencies (e.g., GPT-4 and LlaMA02-7b) in real world diagnostics (22). Just as it is next-to impossible to differentiate a piece of text written through AI from that of a human agent, it will be soon next to impossible to differentiate a synthetically created MRI from that of a real person. Proper regulation of this domain will require participation from funding agencies, ethics review boards, and publishers to ensure we are not marching towards a false flag.

It is clear now that generative AI models are reaching greater complexity and can interact with complex psychological and physiological data and generate a dataset equivalent to modern normative value datasets in a fraction of the time. Their potential for changing the clinical landscape is undoubtably immense and should be pursued. As we attempt to satisfy the immense hunger of such algorithms for data, we need to be mindful of the data being fed through iterations of such models and how that defines the outcomes we observe. Context is everything. Clinical research needs to leverage the power and speed of AI particularly as it relates to vulnerable populations; however, proceeding without caution could lead us to lose the agent and with it, their agency.

## Data Availability

The original contributions presented in the study are included in the article/Supplementary Material, further inquiries can be directed to the corresponding author.
